# Barriers to receiving hepatitis C treatment for people who inject drugs: Myths and evidence

**Published:** 2011-07-01

**Authors:** Peter Higgs, Rachel Sacks-Davis, Judy Gold, Margaret Hellard

**Affiliations:** 1 National Center in HIV Epidemiology and Clinical Research, University of New South Wales, Sydney, Australia; 2 Center for Population Health, Burnet Institute, Melbourne, Australia; 3 School of Public Health and Preventive Medicine, Monash University, Melbourne, Australia

**Keywords:** Hepatitis C virus, Interferon, Drug users, Alcohol, Mental disorder

## Abstract

**Background:**

Alcohol consumption, current injecting drug use, and pre-existing mental illness have been identified as 3 of the main reasons for excluding patients from treatment for hepatitis C.

**Objectives:**

We reviewed the literature to obtain an evidence base for these common exclusion criteria.

**Materials and Methods:**

We reviewed original research and meta-analyses investigating the effects of alcohol consumption, current injecting drug use, and pre-existing mental illness.

**Results:**

We identified 66 study reports relevant to the review, but found only limited evidence to support withholding of treatment on the basis of the 3 previously mentioned exclusion criteria.

**Conclusions:**

Currently, there is a lack of evidence for many of the barriers faced by patients in availing treatment for hepatitis C. Adherence to treat routine was found to be a better predictor of sustained virological response than injecting drug or alcohol consumption during treatment period or the presence of a pre-existing mental disorder. Although several challenges remain, we need to ensure that treatment decisions are based on the best available evidence and the treatment is performed appropriately on a case-by-case basis.

## 1. Background

In developed countries, people who inject drugs (PWID) are at the greatest risk of infection with hepatitis C virus (HCV) [1][2]. Despite the lack of evidence, throughout the 1990s, regulations restricting PWIDs' access to referral for specialist treatment for their chronic HCV infection has existed in a number of western countries [3][4]. The 2005 special issue (40, supplement 5) of Clinical Infectious Diseases was dedicated to HCV infection, managing opiate dependence, and developing models of integrated care for HCV-positive people. The summary in the special issue concluded that there remained important knowledge gaps on the providing the best health-care and treatment for HCV infection in current injecting drug users and stated that further research would be required to specifically address this issue [5].

A recent review on HCV treatment for PWID in patient groups with and without a history of injecting drug use showed that the group with a history of injecting drug use was able to successfully complete treatment with minor variations in treatment outcomes [6]. Presently, a number of international research studies conducted in different treatment settings show that it is possible to successfully provide HCV treatment to current injectors [7][8][9][10]. Previous research has shown that there are individual (both the patient and provider) and structural barriers to HCV treatment [11][12][13]. During a previous literature review on hepatitis C antiviral treatment in PWID, we identified 3 barriers that may on an individual level, affect the treatment in injecting drug users and were commonly cited as the formal exclusion criteria for, or predictors of exclusion from, the antiviral therapy: continued injecting drug use, alcohol use, and pre-existing mental disorders [11][14][15][16][17][18][19][20][21].

## 2. Objectives

Although we observed that individual barriers were not the only ones that prevented injecting drug users from accessing treatment, we limited our review on this to focus more on the above-mentioned 3 barriers. For each barrier, we delineated a myth that underlay the exclusion of affected HCV-positive patients from receiving treatment. In the context of our study, a myth was defined as a commonly held belief that may or may not have been consistent with the available evidence.

## 3. Materials and Methods

A literature review was undertaken on the effects of alcohol consumption, mental disorder, and current injecting drug use on hepatitis C antiviral treatment to evaluate the extent to which the 3 myths were evidence-based. The literature searches were conducted from 2008 to July 2010. By using Ovid MEDLINE®, we searched the entries in the database from 1996 to the present with daily update, and keyword mapping identified the following subject headings to be used as search terms: "hepatitis C," "hepacvirus," "antiviral agents," "alcohol-related disorders," "alcohol drinking," "mood disorders," and "substance use, intravenous". Various combinations and sub-categories of the above subject headings were used in the searchers. We also used the "explode" function in Medline, which allows broad searching of a term while simultaneously narrowing the searches for all other terms in the subject heading. Reference lists in the identified articles were also searched for obtaining any relevant information. We included articles in English that described results from original research or meta-analyses that measure the outcomes of continuous alcohol consumption, a pre-existing mental illness, and/or current injecting drug use in patients receiving hepatitis C antiviral therapy, or articles that reported a successful follow-up for measuring the rates of reinfection of patients who received antiviral therapy.

## 4. Results

Over 400 articles were filtered through the database searches, and 66 were included in this review. In the next section of the paper, we include a critical review of the 3 myths identified.

### 4.1. Myth 1: Illicit use of drugs during HCV treatment may cause complications and increase the chance of reinfection

Few studies, all with small samples (N < 75), have investigated the impact that current injecting drug use during HCV treatment has on the rates of sustained virological response (SVR). Four studies have found similar SVR rates among participants who reported injecting drug during the treatment period and those who had a history of injecting use but had reported no drug use during the treatment [8][10][22][23]. Participants of 2 studies who injected regularly (at least once in every 2 days for a prolonged period during the treatment) were observed to have lower SVR rates than those who abstained from injecting or injected less frequently during the treatment, even though the differences did not reach a statistically significant value [22][23].

Several studies have found that those who continued injecting drug use during treatment were less likely to comply with the treatment regimen and hence did not complete treatment [24][25]. However, 2 other studies showed that frequent injecting drug use during treatment did not affect the probability of attaining an SVR as long as the treatment regimen was completed [8][26]. Few studies have conducted a systematic follow-up of the participants after HCV treatment, and those studies have shown low rates of reinfection [27]. Although there are cases of posttreatment reinfection in the literature [28][29][30], the incidence of such cases is less frequent than the incidence of HCV infection[31] or reinfection [32][33] in community-based studies.

### 4.1.1. Implications

Adherence to treatment appears to be a stronger predictor of whether injectors with chronic HCV infection will achieve SVR than whether or at what levels they inject drugs during the treatment period. The less clear part is how the complex social and environmental factors can be managed so that individuals could have strategies that can help ensure adherence to the treatment regimen. This includes management of side effects that may occur during treatment. Although follow-up after treatment to measure the reinfection rates have been done in a lesser number of participants, to date the reinfection rates that have been observed after treatment are far lower than those observed after spontaneous clearance. Therefore, there is no evidence showing that the potential for posttreatment HCV reinfection is a sound reason for not offering treatment to PWID.

### 4.2. Myth 2: Alcohol consumption before and/or during treatment has major implications for the successful treatment of HCV

The biological mechanisms by which alcohol might affect HCV treatment outcomes have been researched [34][35][36][37]. Alcohol consumption has been found to increase the likelihood of histological steatosis and to accelerate hepatic fibrosis and inflammation in patients with HCV infection [38][39][40][41][42]. Some studies have found that alcohol consumption raises the HCV viral load [41]; however, a meta-analysis of the effect of alcohol consumption on HCV replication failed to show any association between alcohol consumption and HCV viral load [38]. Some studies have reported that a history of alcohol consumption has an adverse effect on HCV treatment outcomes by reducing the probability of attaining SVR [43][44][45][46]. An earlier study had reported a weak dose-response relationship between the quantity of alcohol ever consumed and the likelihood of failing treatment [45]. However, other studies have reported that the weak dose-response effect can be reversed by observing a lengthy period of pretreatment abstinence from alcohol consumption [47][48]. Some other studies have found an association between pretreatment alcohol consumption and treatment outcome; however, a univariate analysis performed after adjusting for other factors in these studies failed to establish any such association [41].

The effect of alcohol consumption during treatment independent of the effect of a history of alcohol consumption, on treatment efficacy remains unclear. Some studies have shown that alcohol consumption during treatment has a negative impact on the treatment outcome moreover, a dose-response effect has been observed during a univariate analysis performed in 1 study [41]. However, some other studies have reported successful treatment outcomes among patients who have continued to consume moderate amounts of alcohol (up to 24 grams per day) during treatment [49][50]. A study in Canada found that participants who had consumed alcohol 6 months prior to treatment were less likely to complete the treatment regimen and were less likely to attain an SVR; however, even in this study, no association could be found between alcohol consumption during treatment and the treatment outcomes [51]. Therefore, in studies where alcohol consumption has been associated with a decrease in SVR, it is unclear if the effect was due to alcohol consumption or a reduced adherence to treatment [40][49]. Indeed, in a treatment study in which continuous alcohol consumption was considered an exclusion criteria for receiving treatment, consuming an average of more than 3 alcoholic drink per day within a year prior to treatment was an independent predictor of early treatment discontinuation after adjusting for factors of race, illicit substance use, and income; however, alcohol consumption itself was not associated with attaining an SVR [52].

Overall, we found that many of the studies on the effect of alcohol on treatment response did not admit heavy drinkers into treatment; therefore, we could only examine the effects of prior alcohol consumption on the treatment outcome [42][43][46][47][48]. Among those studies that did investigate alcohol consumption during treatment, the participant numbers were small, and many studies were on interferon monotherapy, which is a relatively ineffective treatment regimen compared to the latest treatment regimens, and most do not account for potential ambiguity in the outcome [41][45][51].

### 4.2.1. Implications

Until further studies can establish the direct effect of alcohol consumption on treatment success, while adjusting for adherence to HCV treatment, it seems reasonable to advise patients to decrease their level of alcohol consumption before and during HCV treatment. However, given that some patients have successfully completed treatment without abstaining from alcohol consumption, a patient's inability to abstain from alcohol consumption before and during therapy should not be seen as an automatic exclusion criterion for receiving HCV treatment.

### 4.3. Myth 3: Pre-existing mental health problems among PWID lead to HCV treatment being unviable

Depression is one of the most common side-effects of HCV treatment [53]; a review reported that up to 40% of the people being treated with interferon experience a mild to moderate depression [54]. Several studies have investigated the relationship between depression (judging by either a history of depression or a depression score when commencing the treatment) and treatment outcomes. All studies except 1 have observed no significant relationship between depression and treatment adherence [55], treatment completion [55][56][57], early or end of treatment response [55][58], or SVR [56]. A larger study found that patients with depression were significantly less likely to complete treatment; however, there was no significant difference in SVR [59]. One study examined the difference in treatment outcomes between patients with and without schizophrenia and found no difference in the treatment completion or end-of-treatment response, but the patients with schizophrenia were significantly more likely to achieve an SVR [60].

Other studies have involved a combined analysis of various psychiatric disorders on the treatment outcomes. One study found no difference in treatment completion among patients with current or past mood or anxiety disorders [61]. Two studies found no difference in treatment completion, end-of-treatment response, or SVR among patients with a current or past psychiatric disorder and those without [62][63]. One study found no difference in end of treatment response or SVR between patients with a history of mental health issues and/or drug use and those patients without [64]

Notably, 8 of 10 studies listed above involved fewer than 100 individuals, and many studies did not perform multivariate analysis [56][58][59][60][61][62][63][64], were conducted retrospectively [56][60][63][64], and/or had various exclusion criteria relating to ongoing or uncontrolled psychiatric illness [58][59][63][64]. Multiple studies have found an association between a lifetime history of depression, or high depression scores at baseline, and development of depression during HCV treatment [57][59][64][65][66][67]. One study found no difference in the symptoms of depression, schizophrenia, or mania between those with and without schizophrenia at baseline [68]. Similarly, no difference was found in the development of symptoms during treatment when a group of patients with various psychiatric disorders was compared to a group of patients without any psychiatric disorders [69] or when a group of patients with a history of psychiatric disorders and/or illicit drug use was compared to a group of patients without any of these [63]. However, similar to the studies investigating the relationship between pre-existing mental health problems and treatment outcomes, 8 of 11 studies involved fewer than 100 participants, and 2 studies were conducted retrospectively [60][64].

### 4.3.1. Implications

Although, there is some evidence to suggest that having a history of depression or a high depression score when commencing treatment increases the likelihood of an individual developing depression during HCV treatment and potentially affecting treatment completion, there is no evidence to suggest that a pre-existing psychiatric disorder could adversely affect the likelihood of attaining an end-of-treatment response or SVR. Evidence also suggests that people experience a number of side effects from their HCV treatment. These results suggest that rather than automatically excluding individuals with pre-existing psychiatric disorders from treatment, they should receive appropriate care for their psychiatric disorder before, during, and after HCV treatment.

## 5. Conclusion

In most developing countries, formal policies restricting PWID from accessing HCV treatment have been lifted over the past 10 years. Although some PWID now receive treatment, the rates remain low; many PWID are still being excluded from treatment because of concerns about their continuous alcohol consumption, injecting drug use, and pre-existing mental illness. Our review highlights the lack of evidence supporting these exclusions. Many of the studies that we reviewed included only a small number of participants and failed to adjust their findings to account for potential ambiguity. Our evidence suggests that a patient's likelihood of attaining SVR is impacted more by the adherence to treatment than by the frequency of drug injecting during the treatment period. Likewise, although there is evidences showing that a pre-existing mental illness may increase the likelihood of experiencing depression as a side effect of the treatment, from our review, it appears that the patients with pre-existing mental illnesses who complete the treatment are just as likely as other patients to attain SVR; this highlights the role of concurrent management of mental health problems with HCV treatment.

To ensure that alcohol consumption does not have a direct effect on treatment efficacy, continued counseling should be offered to patients on reducing their alcohol consumption before and during treatment. However, as some patients have successfully completed treatment even while continuing to consume moderate amounts of alcohol, alcohol consumption should not be seen as an automatic exclusion criterion for HCV treatment. The decision to treat HCV should be patient centered and made on a case-by-case basis with foremost attention to the needs and interests of the person seeking treatment. The research in this area is ongoing, and as the number of studies increases, we will be able to pool more data on a range of indicators, which may help understand the factor that has the greatest impact on the HCV treatment outcome. At present, there is no compelling evidence for restricting access to PWID seeking HCV treatment. We consider this the right time for making evidence-based decisions rather than making decisions based on pre-conceived ideas about patients who deserve treatment for HCV, and we hope that those involved in deciding to offer HCV treatment would consider the results of our study.
